# Successful secukinumab therapy in plaque psoriasis is associated with altered gut microbiota and related functional changes

**DOI:** 10.3389/fmicb.2023.1227309

**Published:** 2023-08-09

**Authors:** Xueshan Du, Cong Yan, Shuzhen Kong, Delu Che, Bin Peng, Longfei Zhu, Songmei Geng, Kun Guo

**Affiliations:** ^1^Department of Dermatology, The Second Affiliated Hospital of Xi'an Jiaotong University, Xi'an, China; ^2^Center for Dermatology Disease, Precision Medical Institute, Xi'an, China

**Keywords:** secukinumab, gut microbiota, psoriasis, 16S rRNA gene sequencing, shotgun metagenomic analysis

## Abstract

**Introduction:**

The role of gut microbiome dysbiosis in the pathogenesis of psoriasis has gained increasing attention in recent years. Secukinumab, targeting interleukin (IL)-17, has a promising efficacy in psoriasis treatment. However, it remains unclear the gut microbiota alteration and related functional changes caused by successful secukinumab therapy in psoriatic patients.

**Methods:**

In our study, we compared the fecal microbiome profile between psoriatic patients after secukinumab successful treatment (AT) and the other two groups, psoriatic patients without therapy (BT) and healthy people (H), respectively, by using next-generation sequencing targeting 16S ribosomal RNA. Then, shotgun metagenomic sequencing was first used to characterize bacterial gut microbial communities and related functional changes in the AT group.

**Results:**

We found that the diversity and structure of the microbial community in the AT group were significantly changed compared to those in the BT group and the H group. The AT group showed a microbiota profile characterized by increased proportions of the phylum Firmicute, families Ruminococcaceae, and a reduction in the phylum Bacteroidota (elevated F/B ratio). To detect functional alteration, we discovered that secukinumab treatment may construct a more stable homeostasis of the gut microbiome with functional alteration. There were different KEGG pathways, such as the downregulated cardiovascular diseases pathway and the upregulated infectious diseases in the AT group. By metagenomic analysis, the metabolic functional pathway was changed after secukinumab therapy.

**Discussion:**

It seems that gut microbiota investigation during biologic drug treatment is useful for predicting the efficacy and risks of drug treatment in disease.

## 1. Introduction

The microbiome, sometimes called the second genome, refers to a large and diverse community of microorganisms and their genes (Valentini et al., 2021). The human microbiome is defined as the set of micro-organisms that live on or inside our body, including the oral cavity, nostrils, skin, gastrointestinal tract, and genitourinary tract (Opazo et al., 2018; NIH Human Microbiome Portfolio Analysis Team, 2019). The intestinal microbiome has been given an increasing emphasis for the hypothesis on the link between the brain–gut–skin proposed by Stokes and Pillsbury (1930). Due to recent advances in next-generation sequencing, researchers have been able to analyze the biological characteristics of the microbiome and host in diseases, which provides a better understanding of the relationship between the microbiome and the host's health or disease (Clements and Carding, 2018).

Strikingly, several studies have emphasized the vital influence of the gut microbiota on the host immune homeostasis, dysbiosis of which is related to the development of several autoimmune diseases (Benhadou et al., 2018; Myers et al., 2019). The gut microbiome is confirmed to play an important role in modulating both innate and acquired immune responses, alterations of which may lead to an inflammatory process and immune disorders (Palm et al., 2015; Honda and Littman, 2016; Planer et al., 2016). Although the composition of the human gut microbiome becomes relatively stable around 2 years, it can be influenced by numerous factors, including lifestyle, comorbidities, antibiotic courses, and other factors.

Psoriasis, an immune-mediated inflammatory chronic skin disease, affects 2–3% of the population worldwide (Pannu and Rosmarin, 2021; Yang et al., 2021). IL-23/Th17 axis-related inflammatory activation has been reported to be responsible for this pathogeny (Valentini et al., 2021). In the context of genetic predisposition, psoriasis can be triggered by various endogenous and exogenous factors, including bacterial infection, antibiotic treatment, or profound dietary modification (Debbaneh et al., 2014; Di Meglio et al., 2014). Recent studies have demonstrated that the gut microbiome is involved in the underlying pathogenesis of psoriasis since certain gut microbiota have a significant impact on the function of various tissues, including skin (Yan et al., 2017). Actually, many researchers have observed a peculiar composition of gut microbiota in psoriatic patients, especially in the *Firmicutes* and *Bacteroidetes* phyla (Polak et al., 2021).

To date, the traditional treatment for psoriatic patients, including topical and/or systemic therapy, is not adequate all the time. Recently, the advent of immunomodulating biological drugs has led to a substantial improvement in disease control. Secukinumab, a kind of biological drug targeting interleukin (IL)-17, has promising efficacy in psoriasis treatment. Besides, IL-17 inhibitors were reported to be able to alter gut microbiome composition in psoriatic patients (Yeh et al., 2019), which manifested as higher levels of the phylum *Proteobacteria* and lower levels of *Bacteroidetes* and *Firmicutes* by 16S rRNA (Yeh et al., 2019). Furthermore, researchers found that the baseline gut microbiota was significantly different between responders and non-responders to secukinumab treatment, which suggested that the gut microbiota may serve as a potential response to treatment biomarker in psoriasis treated with secukinumab therapy (Yeh et al., 2019). However, metagenomics has not been performed to explore the alteration of the gut microbiome induced by secukinumab therapy in plaque psoriasis, with no exploration of the gut microbiota at the species level and no detailed information on functional gene expression.

Addressing the questions, we collected feces from psoriatic patients without treatment and after successful secukinumab treatment, as well as feces from healthy people as control. By using the 16S rRNA method and shotgun metagenomic analysis, we detected gut microbiota composition and functional change to investigate the influence of successful secukinumab therapy on the gut microbiome in psoriasis.

## 2. Materials and methods

### 2.1. Ethics approval and consent to participate

The authors are accountable for all aspects of the work, ensuring that questions related to the accuracy or integrity of any part of the work are appropriately investigated and resolved. The study was approved by the Medical Ethics Committee of the Second Affiliated Hospital of Xi'an Jiaotong University (2021032). All study subjects signed informed consent forms.

### 2.2. Study subjects

Moderate-to-severe (PASI > 10 or BSA > 10) psoriatic patients successfully treated by 5-month secukinumab treatment (*n* = 11; AT group), which was defined by achieving a PASI 90 response, were selected, and age-, sex-, and disease severity-matched (1:3) psoriatic patients without therapy (BT group) were identified. The control group (H group) compromised age- and sex-matched (1:3 or 1:4) healthy donors (Supplementary Table 1). All participants with 18–25 BMI were selected from the Second Affiliated Hospital of Xi'an Jiaotong University, and they did not receive antibiotics, probiotics, glucocorticoids, or immunosuppression during the 6 previous months. Besides, they have not suffered from diseases of the digestive system, immune system, or other severe illnesses. Finally, we collected 11 samples from the AT group, 32 samples from the BT group (one patient was lost), and 35 samples from the H group for 16S rRNA gene sequencing.

Then 24 samples with high-quality DNA from the AT (8), BT (8), and H (8) groups were selected for shotgun metagenomic sequencing (Supplementary Table 2).

### 2.3. Samples collection and DNA extraction

Fresh fecal samples were collected in a specimen collection kit and stored at −80°C before further manipulation in the laboratory. All fecal samples were transferred to the laboratory in an ice box.

DNA from stool samples was extracted using the DNA Isolation Kit (MoBio, Carlsbad, CA, USA) according to the manufacturer's instructions. Using NanoDrop 2000 (Thermo Fisher Scientific, Waltham, USA), the concentration and purity of bacterial genomic DNA were tested. Total DNA quality was detected by electrophoresis using a 1% agarose gel. All the genomic DNA was stored at −20 and −80°C for short-term and long-term uses, respectively.

### 2.4. The 16S rRNA gene sequencing

The V3 + V4 regions of the 16S rDNA genes were amplified by PCR with the universal primers (F: 5′-ACTCCTACGGGAGGCAGCA-3′, R: 5′-GGACTACHVGGGTWTCTAAT-3′). Next, 16S rDNA gene sequencing was performed on fecal samples from our 78 samples on the Illumina Hiseq 2500 sequencing platform (Biomarker Technologies Corporation, Beijing, China) in PE250 mode (2 × 250 bp paired ends). The analysis of microbiota was carried out on the BMK Cloud platform (www.biocloud.net).

Particularly, the paired-end reads were assembled using the FLASH software to produce Raw Tags. To get clean tags, Trimmomatic (version 0.33) was used to perform a quality filter. Using UCHIME (version 8.1), we screened for and removed putative chimeric sequences. Operational taxonomic units (OTUs) were clustered with more than 97% identity by USEARCH (version 10.0), while the OTUs whose proportions were < 0.005% of the total OTUs were removed.

### 2.5. Shotgun metagenomic sequencing and analysis

A total of 24 samples (*n* = 8 for the AT group, *n* = 8 for the BT group, and *n* = 8 for the H group) were sequenced for the metagenomes. For each sample, 10 ng of genomic DNA was used for PE library preparation with the VAHTS^®^ Universal Plus DNA Library Prep Kit for Illumina. Libraries were sequenced using the Illumina NovaSeq 6000 (PE 150) platform and NovaSeq 6000 S4 Reagent Kit at Biomarker Technologies Corporation (Beijing, China). After being filtered with fastp, the rest of the reads were assembled and predicted with MEGAHIT and MetaGeneMark, respectively. MMseqs2 was utilized to construct non-redundant gene sets at a threshold with a shared sequence identity of over 95% and overlap of over 90%. The metagenomeSeq function of the R software was used to screen different species at different levels. The gene abundance profile was calculated according to Wen et al. (2017). The clean non-redundant gene sequences were queried against the CARD database (RGI model, strict and perfect hits only), the CAZy database (if alignment > 80 amino acids, use *E*-value < 1e-5; otherwise, use *E*-value < 1e-3; a covered fraction of HMM > 0.3), the KEGG database (at the threshold of *E*-value < 1e-5), eggNOG database (at the threshold of *E*-value < 1e-5), and the GO database. We used the R package pheatmap to draw the heat map of these pathways.

### 2.6. Bioinformatics and statistical analysis

Using the Mothur software, alpha diversity based on OTU level, including indices to measure evenness (Simpson) and richness (ACE), Good's coverage, and species accumulation curves, was investigated. The difference in alpha diversity between groups was examined by Student's *t*-test. Using the QIIME software, the Non-MetricMulti-Dimensional Scaling (NMDS) analysis based on binary_jaccard and bray-curtis distances was performed to assess the beta diversity of the bacterial community and functional gene composition, respectively. To test whether beta diversity is statistically different among different samples, permutational analysis of variance (PERMANOVA) was performed using the R software. Linear discriminant analysis (LDA) effect size (LEfSe) was used to further determine the specific significantly different bacterial taxa among cohorts. LDA values > 2.0 with a *p-*value of < 0.05 were considered significantly enriched. The functional abundance spectrum of the Kyoto Encyclopedia of Genes and Genomes (KEGG) ortholog functional profile was predicted by the PICRUSt procedure from the 16S rRNA gene data. Besides, the phenotype prediction of the gut microbiota was analyzed by BugBase using Pairwise Mann-Whitney-Wilcoxon tests. Differential pathway analysis was carried out by metagenomic sequencing based on the Kruskal-Wallis *H*-test and the one-way ANOVA test.

## 3. Results

### 3.1. Characteristics of study participants and sequence of the gut microbiome

We enrolled 78 participants, including 11 psoriatic patients after 5-month secukinumab treatment, 32 psoriatic patients without treatment, and 35 healthy people as controls. There were no significant differences in demographic information between the AT, BT, and H groups. A summary of related characteristics of the above three groups is presented in Table 1, and detailed related information about participants is shown in Supplementary Table 1.

**Table 1:** Characteristics of each group.

	**AT**	**BT**	**H**
Male/female	10/1	29/3	31/4
Age (years)	37.82	38.38	37.31
Types of patients			
Psoriasis vulgaris	11	32	
Psoriasis arthritis	0	0	
Pustular psoriasis	0	0	
Erythrodermic psoriasis	0	0	

AT, psoriasis patients receiving 5-month secukinumab treatment; BT, psoriasis patients receiving traditional therapy instead of biological drugs; H, healthy people.

**Figure 1:**
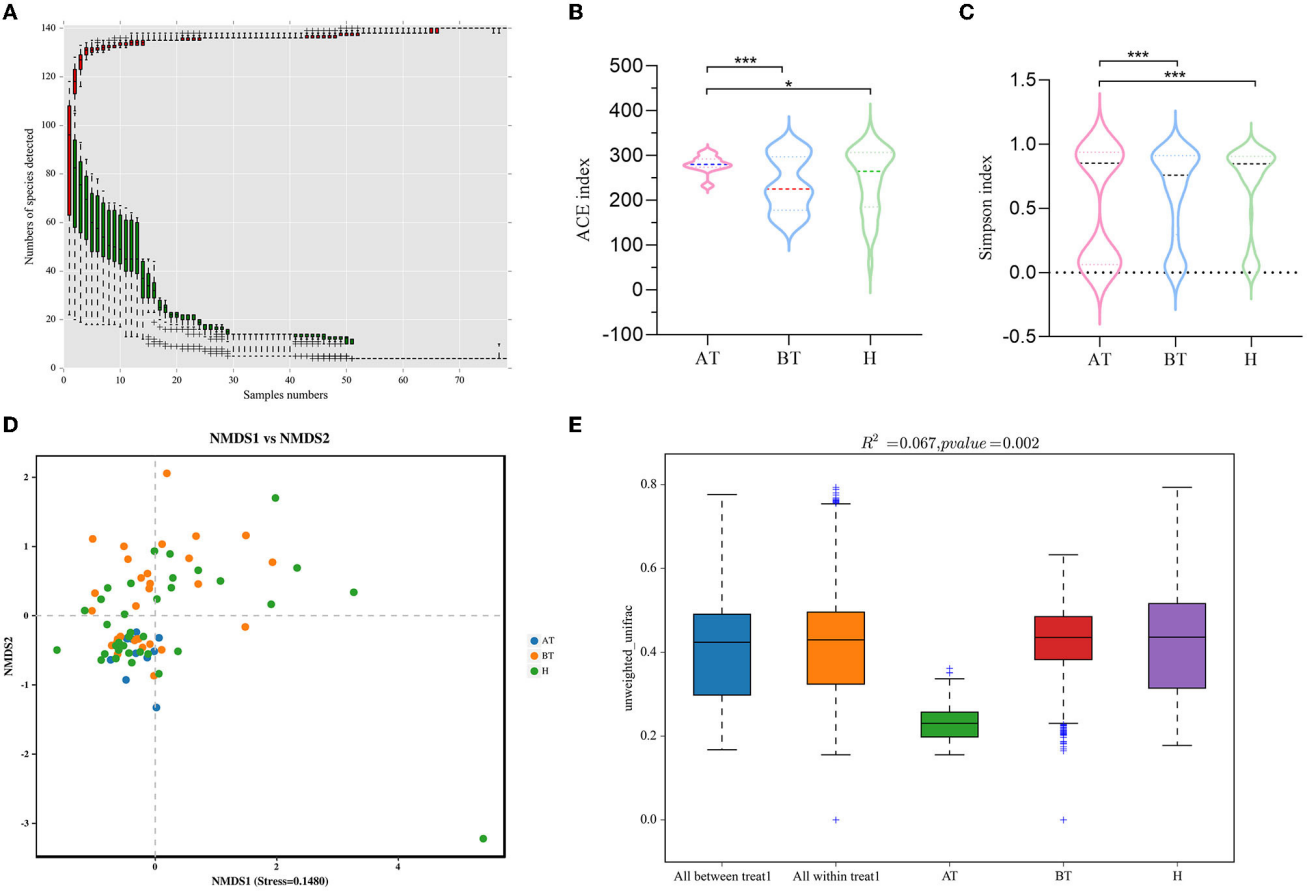
Secukinumab therapy alters gut microbial diversity. **(A)** Species accumulation curves at the genus level had reached a plateau. **(B, C)** The richness of fecal microbiota and structural differences were analyzed by ACE indices **(B)** and Simpson indices **(C)**, respectively. **(D)** The difference in gut microbial communities was investigated using NMDS based on binary_jaccard (Stress < 0.2). **(E)** Permanova analysis based on unweighted_unifrac was used to confirm the significant difference in beta diversity. AT, psoriatic patients after receiving 5-month secukinumab treatment; BT, psoriatic patients without therapy, H, healthy people. ^*^p < 0.05 vs. AT, ^**^p < 0.01, ^***^p < 0.001.

Then, 78 fecal samples were collected, containing 5,841,838 raw sequences with a mean length of 456 bases, for 16S rDNA sequencing and analysis. After quality trimming and chimera checking, we obtained 5,585,894 high-quality sequences in total for further analysis. Four hundred and twenty-four OTUs in all samples were identified, and 382, 412, and 420 OTUs were confirmed in the AT, BT, and H groups, respectively. The data summary is shown in Table 2. All sequences in the three groups had high coverage values of over 99%. The species accumulation curves at the genus level had reached a plateau in all groups (Figure 1A).

**Table 2:** Fecal microbiota community indices.

**Group**	**OTUs**	**Good's**	**Richness index (M ±SD)**	**Diversity index (M ±SD)**
			**ACE**	**Simpson**
AT	382	0.9994	280.9624 ± 6.0319	0.9200 ± 0.0105
BT	412	0.9994	235.4373 ± 10.7863	0.8019 ± 0.0287
H	420	0.9994	246.2601 ± 12.2193	0.8452 ± 0.017

AT, psoriatic patients after receiving 5-month secukinumab treatment; BT, psoriatic patients without therapy; H, healthy people.

For the shotgun metagenomic sequencing, a total of 60.0 GB of data were generated. Also, 5,785,683 genes were predicted using MetaGeneMark. The species accumulation curves at the genus level had reached a plateau in all groups (Supplementary Figure 1).

The above results illustrate that most fecal microbiota species were detected, which provided good sequencing depth for fecal microbiota exploration in the follow-up analysis.

### 3.2. Secukinumab treatment leads to a significant change in gut microbial diversity

We set out to investigate the richness of fecal microbiota and structural differences among these three groups, and the α-diversity (ACE index and Simpson index) of gut microbiota was calculated (Table 2). We discovered that secukinumab therapy induced significantly increased microbiota richness compared to that of the other two groups by ACE indices (Figure 1B) and Simpson indices (Figure 1C). However, there were no significant differences in the α-diversity of gut microbiota between the BT and H groups, as indicated by both the ACE and Simpson indices.

Furthermore, to assess the similarity of gut microbial communities among these three groups, beta diversity was analyzed. NMDS based on binary_jaccard was performed, which suggested that the fecal microbiota of the AT group was different from the other two groups (stress = 0.1480 < 0.2; Figure 1D). The separation trend of the AT and BT groups is more obvious, which is validated significantly (*p* = 0.002, PERMANOVA; Figures 1D, E).

### 3.3. Secukinumab treatment alters gut microbiota composition

**Figure 2:**
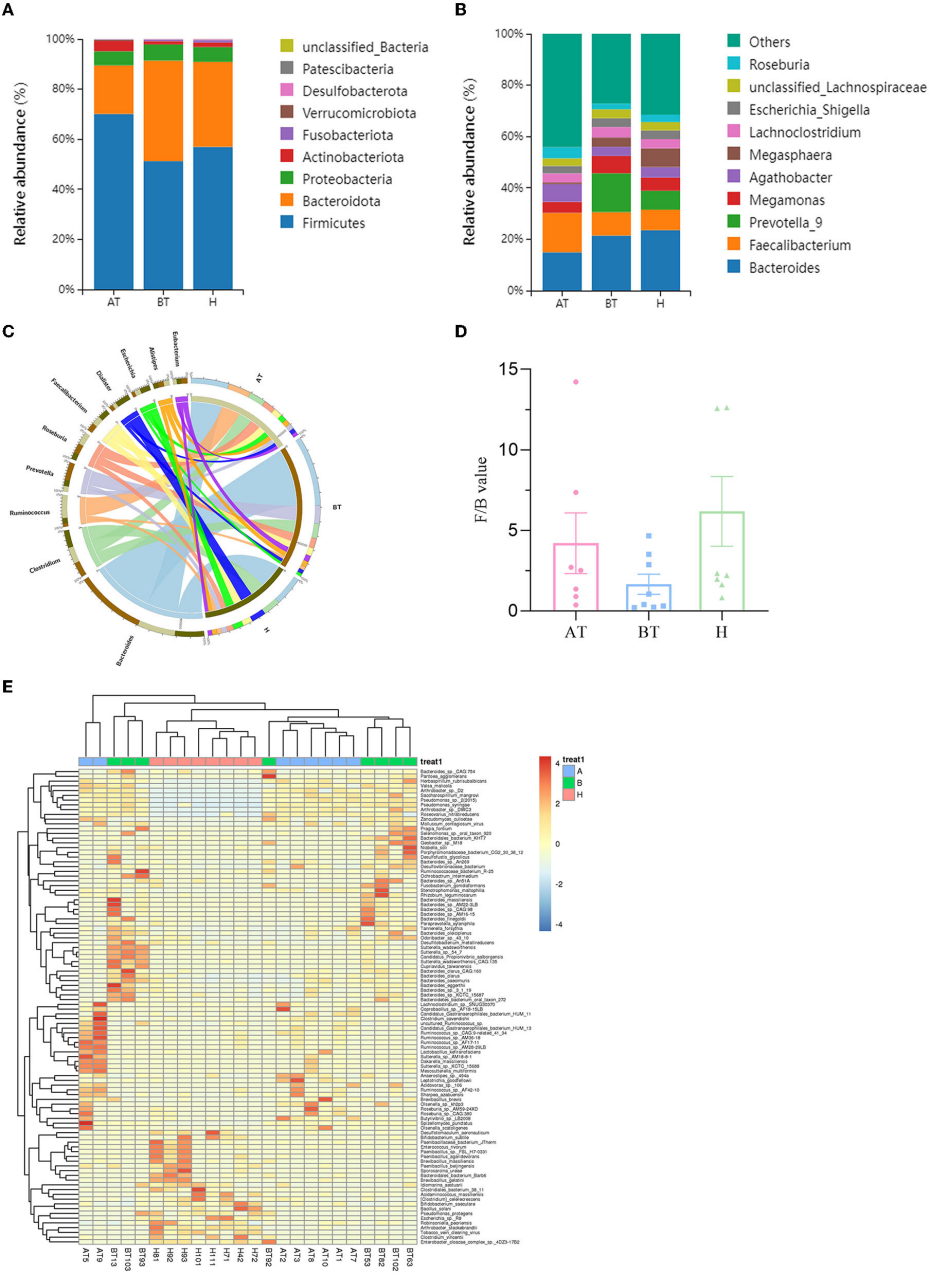
Secukinumab therapy altered microbial community structure at the phylum, family, and genus levels. Relative abundance (%) of the intestinal microbiota determined at the phylum **(A)** and genus **(B)** levels. **(C)** Circos graphs. The left semicircle represents the genera composition of each group. The right semicircle indicates the distribution of each genus in the different groups. **(D)** *Firmicutes/Bacteroides* value. **(E)** Heat map of microbiota composition at the species level. AT, psoriatic patients after receiving 5-month secukinumab treatment; BT, psoriatic patients without therapy; H, healthy people.

The taxonomy of fecal microbiota was evaluated using the RDP classifier. By 16S rRNA gene sequencing, two dominant phyla, namely, *Firmicutes* and *Bacteroidota*, based on different relative abundances among the three groups, were identified. *Firmicutes* (70.12%) increased and *Bacteroidota* (19.38%) decreased in the AT group when compared to the BT group (*Firmicutes* 51.24%, *Bacteroidota* 40.15%) and the H group (*Firmicutes* 56.94%, *Bacteroidota* 33.95%; Figure 2A). At the genus level, the abundance of *Faecalibacterium* (15.31%) was higher, and the abundance of *Bacteroides* (14.91%) and *Prevotella_9* (0.03%) was lower in the AT group than in the other two groups (Figure 2B).

To further explore the microbiota composition, shotgun metagenomic sequencing was used. It is effective to visualize relationships between the microbiota and samples by using the Circos graph. As shown in Figure 2C, the AT group showed a decline in *Bacteroides* and *Prevotella* compared to the BT group and a decrease in *Dialister* compared to the H group at the genus level. But the level of *Ruminococcus* was higher in the AT group. The changes in the rest of the groups were unnoticeable (Figure 2C). *Bacteroides* and *Firmicutes* were predominant in the gut microbiota of all subjects. *Firmicutes/Bacteroides* values were higher in the AT group than in the BT group, as shown in Figure 2D. At the species level, Figure 2E shows a heat map of microbiota composition based on the one-way ANOVA test. More detailed information on microbiota composition at the phylum, family, and taxonomy levels is shown in Supplementary Figures 2–4.

To further evaluate the differences in the gut microbiota composition between the two groups, a metastatistical analysis was performed. On a phylum level, there was a significant increase in *Firmicutes* and a decrease in *Bacteroidota* in the AT group compared to the other two groups (Table 3). At the genus level, when compared to the H group, the AT group presented obviously decreased genera *Bradyrhizobium, unclassified_Firmicutes* and *uncultured_Clostridiales_bacterium*, but increased genera *Hydrogenophaga, Pantoea*, and *unclassified_Comamonadaceae* (Table 3). In addition, when compared to the BT group, the AT group showed statistically decreased genera *Bradyrhizobium, Hydrogenophaga*, and *Lactococcus* but increased genera *Pantoea* and *unclassified_Comamonadaceae* (Table 3).

**Table 3:** Taxonomic differences among three groups at the phylum (A) and genus (B) levels.

	**AT**	**H**	**BT**
**(A)**			
p: Firmicutes	7.00E-01 ± 3.27E-02	5.37E-01 ± 3.52E-02^*^	4.98E-01 ± 4.14E-02^**^
p: Bacteroidota	1.96E-01 ± 2.99E-02	3.67E-01 ± 3.49E-02^*^	4.16E-01 ± 4.33E-02^**^
**(B)**			
g: Bradyrhizobium	2.01E-04 ± 2.42E-05	4.96E-05 ± 1.35E-05^*^	5.56E-05 ± 2.74E-05^*^
g: Hydrogenophaga	3.21E-04 ± 3.74E-05	1.47E-05 ± 1.38E-05^*^	3.57E-05 ± 1.81E-05^*^
g: Lactococcus	0		2.16E-04 ± 2.10E-04^*^
g: Pantoea	8.44E-04 ± 7.63E-05	3.17E-04 ± 7.39E-05^*^	3.01E-04 ± 7.84E-05^*^
g: unclassified_Comamonadaceae	6.36E-04 ± 1.08E-04	2.81E-05 ± 2.29E-05^*^	3.75E-05 ± 1.83E-05^*^
g: unclassified_Firmicutes	0	3.60E-05 ± 3.60E-05^*^	
g: uncultured_Clostridiales_bacterium	0	3.04E-04 ± 3.04E-04^*^	0^#^
g: Alloprevotella		4.14E-04 ± 4.04E-04	0^#^
g: Butyrivibrio		2.33E-03 ± 2.33E-03	0^#^
g: Prevotella_7		1.20E-06 ± 8.88E-07	9.62E-04 ± 8.75E-04^#^

Values are expressed as means ± SD.

P.adj indicates the P-values corrected by multiple testing. ^*^p.adj < 0.05 vs. AT, ^**^p.adj < 0.01 vs. AT, ^#^p.adj < 0.05 vs. H.

AT, psoriatic patients after receiving 5-month secukinumab treatment; BT, psoriatic patients without therapy; H, healthy people.

**Figure 3:**
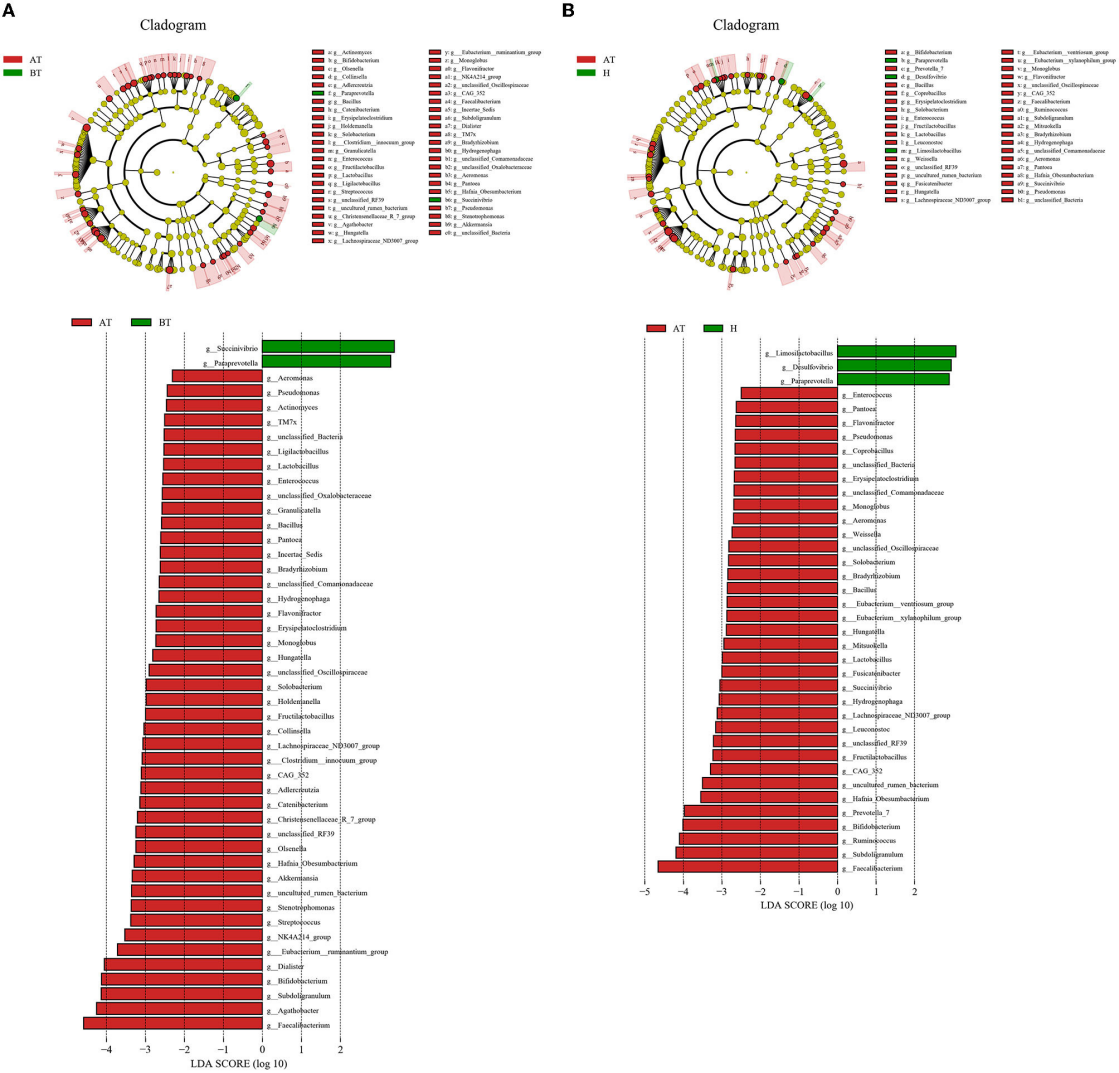
The significant difference in bacterial taxa induced by secukinumab therapy. **(A, B)** Cladogram view of the representative microbial structure among the three groups. A phylogenetic view of the representative microbiota for each clinical group. The dominant microbial classes for the specific groups are represented by different colors. A taxonomic cladogram obtained from LEfSe analysis indicates the phylogenetic distribution of the gut microbiota of the AT group and the BT group **(A)**, and the H group **(B)** at the genus level. Histogram of LDA scores to demonstrate the effect size and rank of differentially abundant taxa (LDA score > 2.0). AT, psoriatic patients after receiving 5-month secukinumab treatment; BT, psoriatic patients without therapy; H, healthy people.

Consequently, we investigated the specific significantly different bacterial taxa among these three groups by using LEfSe analysis at the genus level (LDA score > 2, *p* < 0.05). Several genera, including *Faecalibacterium, Agathobacter, Subdoligrnulum, Bifidobacterium*, and *Dialister*, were significantly enriched in the AT group, while the genera *Succinivibrio* and *Paraprevotella* were obviously enriched in the BT group (Figure 3A). Besides, we detected an alteration in gut microbiota composition between the AT group and the H group. We identified that genera, such as *Faecalibacterium, Subdoligrnulum*, and *Ruminococcus*, were significantly increased in the AT group, whereas *Limosilactobacillus, Desulfovibrio*, and *Paraprevotella* were significantly enriched in the H group (Figure 3B). Finally, in Supplementary Figure 5, we discovered that the genera *Prevotella_7, Weissella*, and *Lactococcus* were increased in the BT group, while *Megasphaera, Dialister, Romboutsia*, and other genera were enriched in the H group.

### 3.4. Secukinumab treatment alters the phenotypic functions of the gut microbiota in psoriatic patients

**Figure 4:**
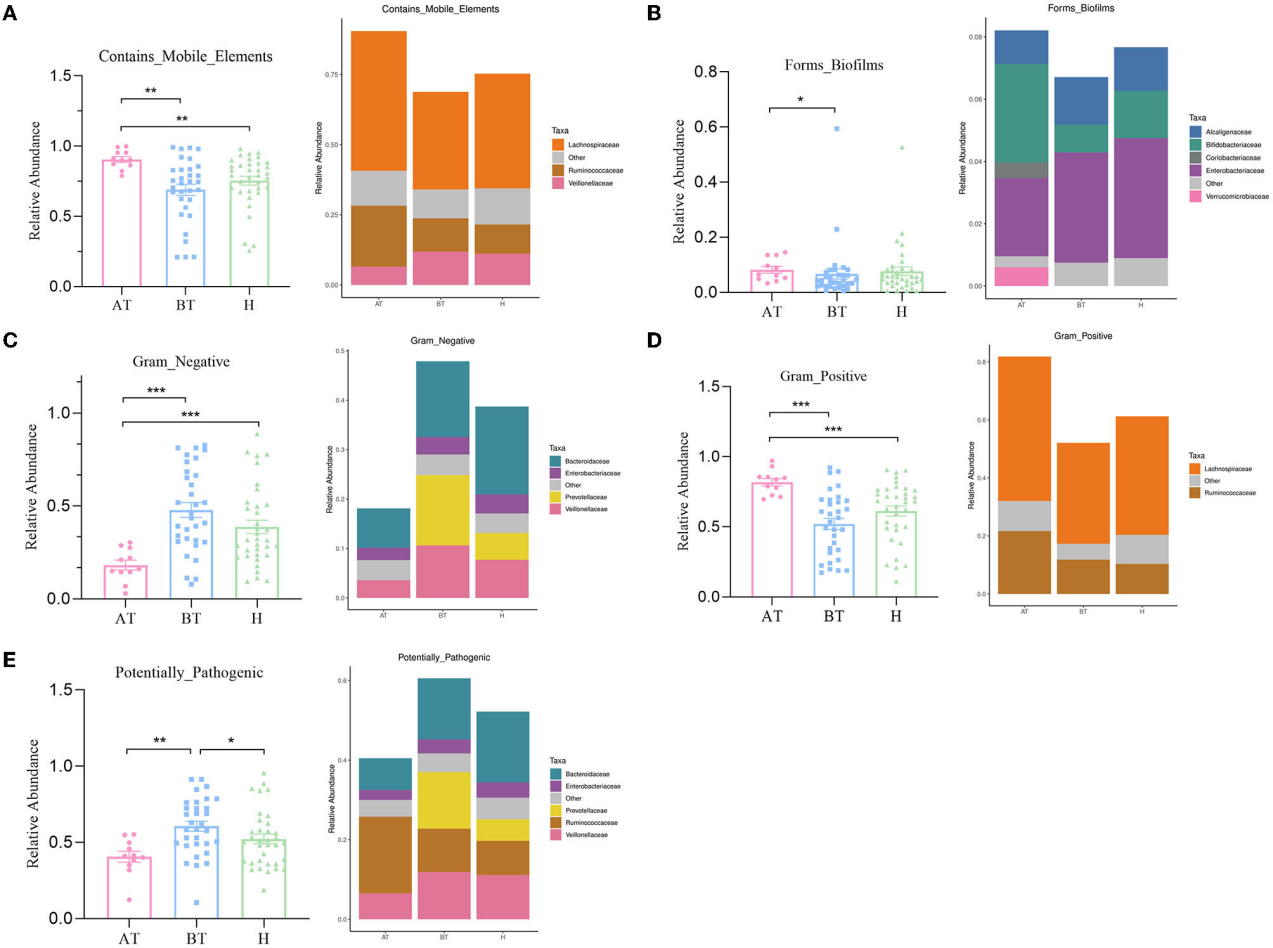
The potential prediction for phenotypic functions of gut microbiota after secukinumab therapy. Five predicted phenotypic functions by BugBase, including containing mobile elements **(A)**, forming biofilms **(B)**, gram-negative **(C)**, gram-positive **(D)**, and potentially pathogenic **(E)**, have been found to be significantly changed after 5-month secukinumab therapy. AT, psoriatic patients after receiving 5-month secukinumab treatment; BT, psoriatic patients without therapy; H, healthy people. ^*^p < 0.05 vs. AT, ^**^p < 0.01, ^***^p < 0.001.

The potential prediction for phenotypic functions in complex communities among these three groups was analyzed by BugBase. Five predicted phenotypic functions (contains mobile elements, forms biofilms, gram-negative, gram-positive, and potentially pathogenic) exhibited significant differences in abundance (Figure 4). The AT group showed an obviously increased abundance in the mobile elements phenotype than the other two groups, which manifested as increased *Lachnospiraceae* abundance and *Ruminococcaceae* but decreased *Veillonellaceae* abundance (Figure 4A). Besides, the gut microbiota of the AT group demonstrated a more significant abundance in forming biofilms phenotype, having an elevated abundance of *Bifidobacteriaceae, Coriobacteriaceae*, and *Verrucomicrobiaceae*, and reduced abundance of *Enterobacteriaceae* and *Alcaligenaceae* than the H group (Figure 4B). Furthermore, we observed that the abundance of gut microbiota in the AT group was significantly lower in gram-negative phenotype (*Veillonellaceae, Prevotellaceae, Enterobacteriaceae*, and *Bacteroidaceae*; Figure 4C), while a statistically higher abundance of gut microbiota in the gram-positive phenotype (*Lachnospiraceae* and *Ruminococcaceae*) was confirmed than in the other two groups (Figure 4D). Finally, a potentially pathogenic phenotype was evaluated. The gut microbiota abundance of the AT group and the H group was obviously reduced in this phenotype compared to the BT group. The AT group suggested massively decreased *Veillonellaceae, Prevotellaceae*, and *Bacteroidaceae*, while the H group demonstrated obviously reduced *Prevotellaceae* and *Bacteroidaceae* (Figure 4E).

### 3.5. Secukinumab therapy influenced the biological function of the gut microbiota in psoriatic patients

To better understand how biological functions in secukinumab treatment may be affected, we used PICRUSt2 to evaluate the composition of functional genes in the fecal microbiota of our samples. As a result, a total of five significantly different functional COGs between the AT group and the H group were found, as well as seven COGs between the AT group and the BT group were predicted (Supplementary Figure 6).

**Figure 5:**
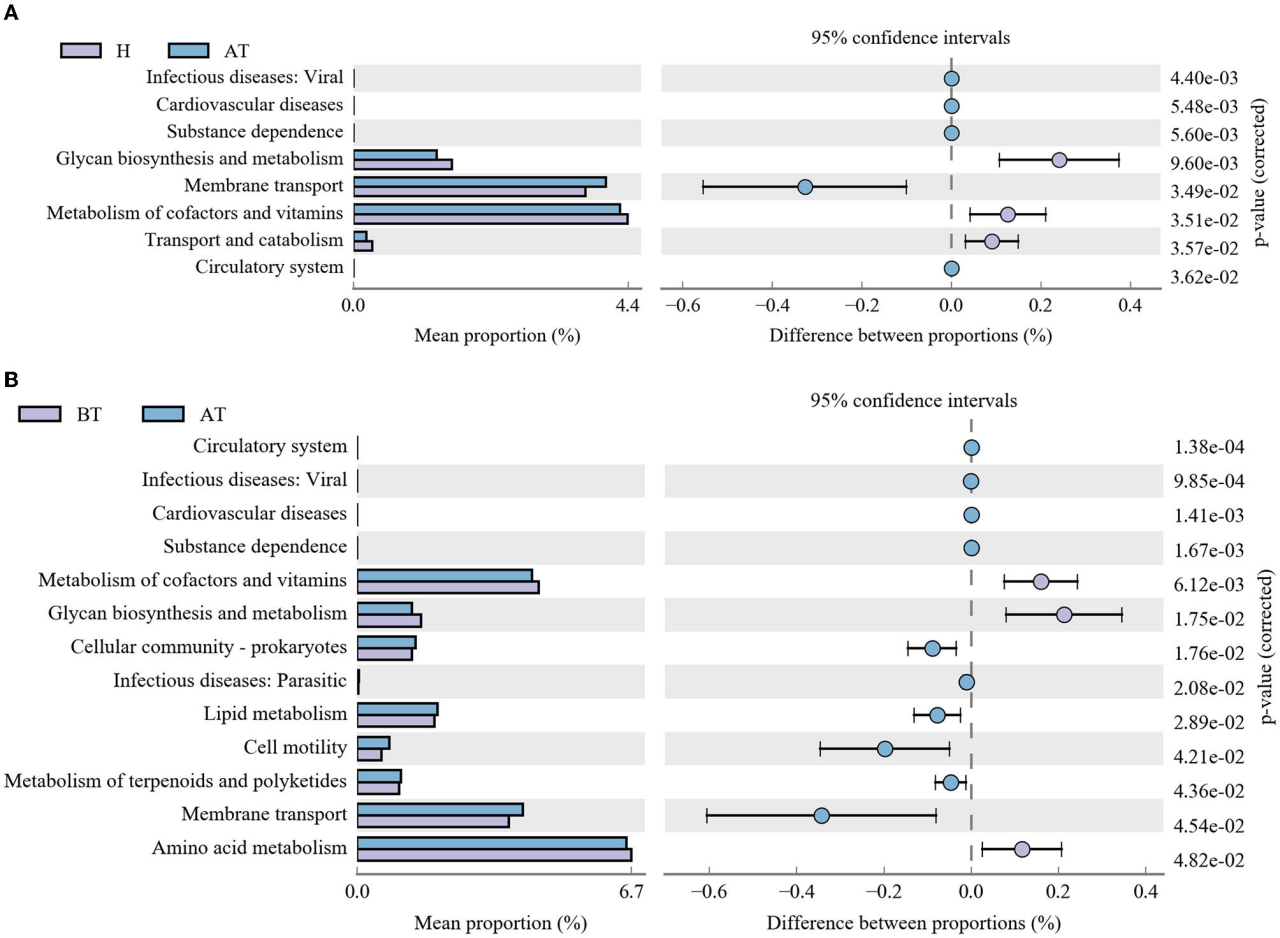
Functional capability analysis based on the mean abundances of KEGG pathways by using PICRUSt. **(A)** Comparison of KEGG pathways between the AT group and the H group. **(B)** Functional differences in KEGG pathways between the AT group and the BT group. AT, psoriatic patients after receiving 5-month secukinumab treatment; BT, psoriatic patients without therapy; H, healthy people.

Moreover, further analysis in the context of the KEGG database was performed to better clarify metabolic function changes in the community samples. In the AT group, the functional genes for the metabolism of terpenoids and polyketides, membrane transport, substance dependence, and infectious diseases: viral were highly enriched, whereas metabolism of cofactors and vitamins, glycan biosynthesis and metabolism, transport and catabolism, and the circulatory system were significantly reduced in comparison with the H group (Figure 5A). Detailed information is shown in Supplementary Table 3A. Additionally, compared to the BT group, eight KEGG pathways (including infectious diseases, such as viral, and infectious diseases, such as parasitic, substance dependence, and others) were significantly enriched and five KEGG pathways (cardiovascular diseases, circulatory system, glycan biosynthesis and metabolism, amino acid metabolism, and metabolism of cofactors and vitamins) were statistically reduced in the AT group (Figure 5B and Supplementary Table 3B).

### 3.6. Shotgun metagenomic functional analysis

**Figure 6:**
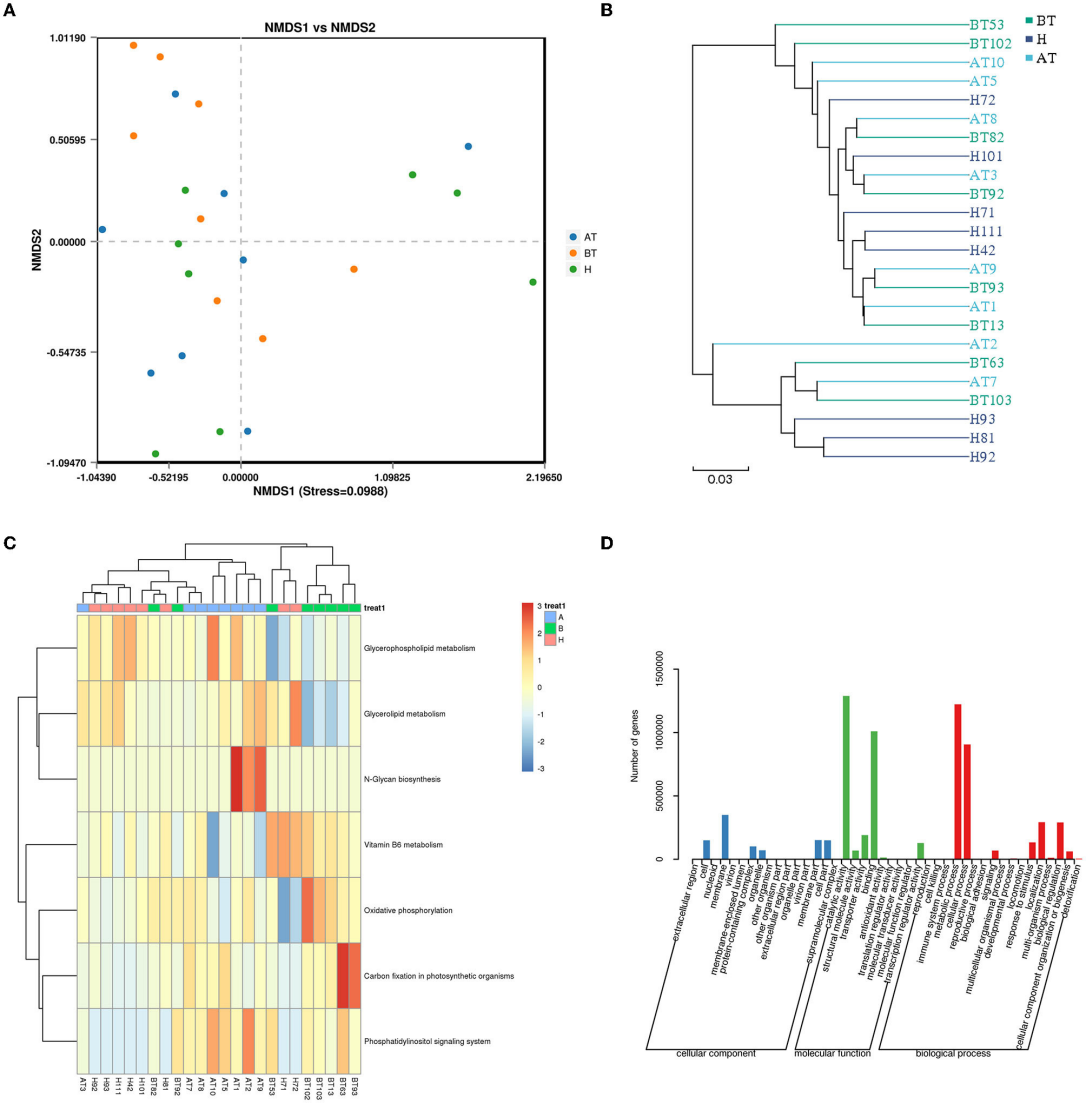
Functional capability analysis for shotgun metagenomic sequencing based on the mean abundances of KEGG pathways. **(A)** The difference in gut microbial communities was investigated using NMDS based on binary_jaccard (Stress < 0.2). **(B)** UPGMA method of three groups. **(C)** Heat map of the KEGG pathway based on the one-way ANOVA test. **(D)** GO analysis. AT, psoriatic patients after receiving 5-month secukinumab treatment; BT, psoriatic patients without therapy; H, healthy people.

To further identify functional changes that occur in response to secukinumab therapy, shotgun metagenomic analysis was also used. NMDS based on bray_curtis was performed, which suggested that the fecal microbiota of the BT group was different from the other two groups (stress = 0.0988 < 0.2; Figure 6A). As shown in Figure 6B, the UPGMA method of beta diversity generated two major clusters.

Moreover, to better clarify metabolic function changes in samples, the KEGG database was used. Pathways that changed in the AT group were related to upregulated glycerophospholipid metabolism, glycerolipid metabolism, N-glycan biosynthesis, and the phosphatidylinositol signaling system (Figure 6C). In the BT group, KEGG metabolic pathways indicated that the gut microbiome was enriched in oxidative phosphorylation and carbon fixation in photosynthetic organisms but decreased in glycerophospholipid metabolism and glycerolipid metabolism (Figure 6C). Interestingly, the KEGG metabolic pathways of the H group also showed higher expression of glycerophospholipid metabolism and glycerolipid metabolism, which is similar to that of the secukinumab therapy group (Figure 6C).

GO analysis showed that following treatment with secukinumab, metabolic process and cellular process were the dominant terms in the biological process category, whereas structural molecule activity and binding were the most enriched in the molecular function category (Figure 6D). More detailed information and a heat map on the CARD database (Supplementary Figure 7), eggNOG database (Supplementary Figure 8), and CAZy database (Supplementary Figure 9) are shown in Supplementary material.

## 4. Discussion

Secukinumab treatment alters the profile of the microbiota. Manasson et al. reported IL-17 inhibitors-induced bacterial and fungal perturbations in psoriatic arthritis (PsA)/spondyloarthritis (SpA) patients, which were more characterized by significant changes in *Clostridiales* and related taxa (Manasson et al., 2020). In psoriasis patients treated with IL-17 inhibitors, levels of *Bacteroides stercoris* and *Parabacteroides merdae* were significantly increased at week 24, while those of *Blautia* and *Roseburia* were significantly reduced by 16S rRNA gene sequencing (Huang et al., 2023). In this study, we investigated the composition of the gut microbiota in psoriatic patients after successful secukinumab therapy by 16S rRNA gene sequencing. Furthermore, it is the first report to explore the gut microbiome alteration and functional change induced by secukinumab in plaque psoriatic patients by shotgun metagenomic analysis. Our results indicated that successful secukinumab therapy caused significantly elevated microbiota richness and changed biodiversity, as well as an alteration in the composition of the gut microbiota, which may modulate the inflammatory reaction in psoriasis (Rizzatti et al., 2017). When compared to psoriasis without therapy, the psoriatic patients with successful secukinumab therapy showed a microbiota profile characterized by increased proportions of the phylum *Firmicute*, genera *Pantoea* and *unclassified_Comamonadaceae*, but decreased phylum *Bacteroidota* and genera *Bradyrhizobium, Hydrogenophaga*, and *Lactococcus*.

It has been confirmed that the pathogenesis of psoriasis is associated with a systemic immune and inflammatory response, which could alter gut mucosa and thereby induce gut inflammation (Yegorov et al., 2020). The *Firmicutes/Bacteroides* (F/B) ratio has been emphasized a lot before in the gut microbiome of psoriasis (Hidalgo-Cantabrana et al., 2019; Shapiro et al., 2019; Dei-Cas et al., 2020). Chen et al. (2018) have found a distinct fecal microbial community structure in psoriasis patients, with an increased abundance of the phylum *Firmicutes* and a decreased abundance of the phylum *Bacteroidetes* across different subgroups of subjects. In psoriasis patients treated with IL-17 inhibitors, Huang YH demonstrated that levels of *Bacteroides stercoris* were significantly increased (Huang et al., 2023). Both *Firmicutes* and *Bacteroidetes* are well-known short-chain fatty acid (SCFAs) producers, including acetate, propionate, and butyrate (Den Besten et al., 2013). Acetate and propionate are mainly produced by the phylum *Bacteroidetes*, while butyrate is produced by the phylum *Firmicutes* (Den Besten et al., 2013). Butyrate is a vital factor in maintaining the epithelial barrier, leading to anti-inflammatory effects. Also, it could suppress oxidative stress and regulate the balance between Th17/Treg lymphocytes (Zeng et al., 2017; Myers et al., 2019; Polak et al., 2021). We found that the F/B ratio is higher in the gut microbiota of psoriasis patients after secukinumab therapy, which causes increased butyrate synthesis (Perry et al., 2016; Komaroff, 2017). We speculated that secukinumab therapy enriched the gut microbiome and F/B ratio in psoriatic patients, which may modulate gut dysbiosis in psoriasis by metabolites and have evident anti-inflammatory effects.

In addition, we found higher proportions of *Ruminococcaceae* in the secukinumab therapy group. *Ruminococcaceae* has been confirmed to be correlated with the number of medium-chain fatty acids (MCFAs), which support Th1 and Th17 cell differentiation (Scher et al., 2015; Haghikia et al., 2016). Th17 cells, which produce the essential proinflammatory cytokine interleukin-17, play a central role in psoriasis, while Th1 cells participate in the occurrence of disease. The interactions between Th1/Th17 cells and dendritic cells, mast cells, macrophages, and neutrophils promote the inflammatory response via IL-17, IL-19, IL-22, IL-23, TNF-α, and other inflammatory cytokines, which results in the formation of psoriatic plaques (Clements and Carding, 2018; Polak et al., 2021). As the MCFAs modulate Th1/Th17 differentiation, the upregulated concentration of MCFAs related to the increased abundance of the family *Ruminococcaceae* should help improve the course of psoriasis.

Furthermore, we explored the gut microbiota-related functional change after secukinumab therapy. By the BugBase analysis, we discovered that the secukinumab treatment group exhibited more significant abundance in containing mobile elements phenotype, forming biofilms phenotype, and gram-positive phenotype and less abundance in gram-negative phenotype and potentially pathogenic phenotype. Biofilms are described as a complex structure of the microbiome that can grow on many different surfaces, the formation of which can promote pathogenicity as well as the stability of bacterial communities, leading to stronger stress tolerance (Mamphogoro et al., 2021). Mobile genetic elements (MGEs) contribute to bacterial adaptation and evolution, which improve resistance to environmental change (Durrant et al., 2020). Therefore, we suggested that secukinumab therapy constructs a more stable gut microbiome. Finally, the potentially pathogenic phenotype of the gut microbiota was reduced after secukinumab therapy in psoriasis.

Using the COGs and KEGG pathway analysis, we found that three KEGG pathways, including the metabolism of cofactors and vitamins, glycan biosynthesis and metabolism, and circulatory system, were significantly reduced in the secukinumab therapy group compared to the other two groups. To further assess the metabolic functional pathway, shotgun metagenomic functional analysis showed that the KEGG pathway of secukinumab therapy was enriched in glycerophospholipid metabolism and glycerolipid metabolism, which was similar to healthy control. Moreover, GO analysis showed the metabolic process was the dominant term in the biological process category in the secukinumab therapy group. Psoriasis is related to an increased risk of developing cardiovascular disease and metabolic syndrome (including atherosclerosis, obesity, diabetes, hyperuricemia, etc.; Takeshita et al., 2017; Wu et al., 2022). Our study suggests that secukinumab therapy affects the metabolic pathways in psoriasis. Some studies found that secukinumab treatment resulted in an improvement of serum lipids and showed neutral to favorable long-term trends in metabolic syndrome (Gerdes et al., 2020; Piros É et al., 2021). The impacts of the systemic biologics used to treat psoriasis on the incidence and progression of metabolic syndrome are under investigation.

KEGG pathways of infectious diseases: parasitic and viral were significantly increased after secukinumab therapy in psoriasis. Actually, infection is the most reported side effect of secukinumab therapy, which is in accordance with our data on gut microbiota (Ergun et al., 2021). It should be given more attention when using secukinumab treatment.

The limitations of the study are as follows. (1) Only Chinese patients were included in this study, and more patients of different races are needed. (2) The proportion of female patients was lower in our study, and future research should take the gender ratio into consideration. (3) The dietary habit bias cannot be totally limited, which may have an impact on the gut microbiota. (4) The potential mechanism of how secukinumab regulates the gut microbiome has not been detected, which deserves to be further explored.

## 5. Conclusion

The findings in our study reveal that secukinumab enhances the richness and changes the diversity of the gut microbiota, which manifests as an alteration of gut microbiome composition. A more stable gut microbiota with less potential pathogenicity was constructed after secukinumab therapy. Besides, secukinumab treatment causes metabolic functional pathway changes as well as other functional gene expression changes. In future studies, more information on gut microbiota investigation during biological drug therapy deserves to be added, which is a vital way of predicting the efficacy and risks of drug treatment.

## Data availability statement

The original contributions presented in the study are publicly available. This data can be found here: https://www.ncbi.nlm.nih.gov/geo/query/acc.cgi?acc=GSE239722, accession number GSE239722.

## Ethics statement

The studies involving human participants were reviewed and approved by Medical Ethics Committee of the Second Affiliated Hospital of Xi'an Jiaotong University (2021032). The patients/participants provided their written informed consent to participate in this study. Written informed consent was obtained from the individual(s) for the publication of any potentially identifiable images or data included in this article.

## Author contributions

XD, KG, SG, and CY were involved in the conception. XD and DC provided the study design. SK and BP assembled the data. CY performed analyses with contributions from SK, BP, LZ, and DC. XD, SG, and CY provided data visualizations. XD wrote the manuscript. KG revised the article. All authors read and approved the final manuscript.
